# Choice and perception of the nursing profession from the perspective of Polish nursing students: a focus group study

**DOI:** 10.1186/s12909-016-0765-3

**Published:** 2016-09-20

**Authors:** Ludmila Marcinowicz, Anna Owlasiuk, Barbara Slusarska, Danuta Zarzycka, Teresa Pawlikowska

**Affiliations:** 1 Department of Primary Health Care, Medical University of Bialystok, Mieszka I 4 B, 15 054 Bialystok, Poland; 2 Chair of Community Nursing, Department of Oncology and Environmental Health Care, Faculty of Health Sciences, Medical University of Lublin, Lublin, Poland; 3 Department of Paediatric Nursing, Faculty of Health Sciences, Medical University of Lublin, Lublin, Poland; 4 Health Professions Education Centre, Royal College of Surgeons in Ireland, Dublin, Ireland

**Keywords:** Nursing profession, Nursing students, Career, Qualitative study, Focus group

## Abstract

**Background:**

Although previous quantitative studies provide important information on the factors which influence the choice of nursing as a career, qualitative analysis makes it possible to study the subject more thoroughly. The purpose of this study was to conduct an in-depth analysis of the reasons why Polish students choose nursing as a profession and their later perception of the job based on experiences acquired during the nursing course.

**Methods:**

A qualitative descriptive study was designed. We organized 8 focus group discussions with third-year nursing students. A total of 76 students participated in the study.

**Results:**

Several reasons why students had chosen the nursing profession were identified: desire to help others, family tradition, desire to work abroad, failure to get into another course, pure chance, and low admission requirements (relative to medical studies). The participants’ views of the nursing profession were based on their own personal experiences or observations of nurses at work. Often these observations were superficial, concerning only selected fragments of nursing work. The participants also identified reasons for there being low regard for the nursing profession.

**Conclusion:**

The decision about choosing nursing is mainly determined by practical aspects, e.g., the opportunity for employment. Although young people are aware of the low prestige of the nursing profession in Poland, they believe it is possible to improve its image and enhance its prestige.

**Electronic supplementary material:**

The online version of this article (doi:10.1186/s12909-016-0765-3) contains supplementary material, which is available to authorized users.

## Background

The nursing profession has historically been undervalued in Poland, but in recent years it has been given higher regard. In an occupational prestige ranking published in 2013, nurses ranked sixth (behind firefighter, university professor, qualified worker, miner, and factory engineer) [1]. However, nurses’ self-evaluation of their profession is lower than the valuation given to it by patients and other medical practitioners, including doctors [2]. Experts suggest that the recent elevation of the nursing profession may be related to the considerable improvement in nurses’ qualification level, i.e., university education [3]. The current model of nursing education in Poland (in use since the year 2000), conforms to the Bologna Process and European Union (EU) directives and involves a two-cycle system. The first cycle (3 years) ends with a Bachelor’s Degree in Nursing. The second cycle (2 years) leads to a Master’s Degree in Nursing. In accordance with the educational standard, a 1st cycle university course must involve at least 4720 h, including 1100 h of practical classes and 1200 h of professional traineeship [4].

Young people’s interest in the nursing profession is not high, and a Polish medical journal referred to nursing as a “dying profession” [5]. Apart from low pay, which is still below the country’s average [3], there are insufficient numbers of nurses in Poland [6], which is an additional stressor on the profession [7]. Against the background of other Organisation for Economic Co-operation and Development (OECD) countries, the ratio of nurses per 1000 residents in Poland is low: in 2011 it was 5.2, while in Switzerland it was 16.6 [8]. Another problem is the rising mean age of the people working as nurses. Analyses by the Head Chamber of Nurses and Midwives showed that in 2008 the mean age of nurses in Poland was 44.2, and in 2013 it was 48.7 [6]. Nurses’ dissatisfaction with their job, especially with the remuneration, is manifested by labor strikes [9]. Survey-based research carried out among 150 nurses and 150 hospitalized patients showed that patients (86 %) and nurses (56 %) generally support nurses’ strikes. The main reasons why such strikes are organized, in the opinion of nurses, are: to achieve higher wages, to improve working conditions, and to improve the image of the nursing profession [9].

### Literature review

It is important to ascertain how students perceive nursing and why they decide on nursing as a career. Reasons for becoming a nurse are a subject of interest for researchers, who apply both quantitative and qualitative approaches when studying them. The desire to help or care for others is reported by researchers in many countries [10–15]. Other reasons for becoming a nurse are the availability of training close to home, pure chance, recommendation from family and friends, and not being able to get into any other study program [12]. A study by Mooney et al. [16] found that the main reason for the choice of nursing as a career was the job situation it offered, explained as job security, easy access to jobs, and diverse opportunities in the career. ‘Job situation’ came out on top, because students sought an occupation in which they would not likely become unemployed, as nursing was perceived as favorable for its ability to provide security of employment and a steady income. Other authors found reasons to pursue nursing, such as personal aspirations (e.g., enjoyment or love of nursing) and career aspirations (e.g., the ability to enter tertiary education) [14]. Bahraini nursing students perceived nursing as providing care, helping people, and a humanitarian vocation; however, nursing in that country was considered not to have much social approval, with cultural issues impacting on the values attached to nursing as a career choice [17]. Research outcomes in psychology show that the original job choice is related to subsequent job satisfaction and commitment [18].

Recent Polish research established that the reasons young people chose nursing as a career are similar to those in other countries, including the desire to help people, interest in medical sciences, availability of job opportunities after graduation, the desire to receive higher education, family tradition, failure to get in to other university courses, and pure chance [19–21]. According to other authors, motivations for the choice of nursing education are mixed, but pro-community motivations, focused on the good of others, prevail [22]. The results of survey-based research of professionally active nurses in Poland show that most of them chose their career mainly out of the wish to help others and to feel useful [23].

Although previous quantitative studies provide important information on the choice of nursing as a career [24], qualitative analyses would make it possible to study the subject more thoroughly. Indeed, at least some authors of recent quantitative studies also see the need for further qualitative analyses [11]. Moreover, an important aspect justifying the need for in-depth analysis of the subject is provided by the research of Franek et al. [25] on interest in the nursing profession among 100 Polish secondary school students, which showed only limited interest in the occupation: only one out of five participants showed any interest in this university course, and only one in ten actually wanted to enter the profession. Thus, it is important to study the subject in terms of the new generation of nurses-to-be and the new social reality in Poland. The aim of the research was to undertake an in-depth analysis of the reasons why students choose the nursing profession, and the perception of the job under the influence of experiences acquired during nursing school.

## Methods

A qualitative descriptive design using focus groups was adopted in this study as the method of choice when straight descriptions of phenomena are desired. Qualitative description is especially amenable to obtaining frank and largely unadorned, i.e. minimally theorized, answers to research questions [26]. This methodological approach was deemed the most suitable for analyzing the reasons why the participants in our study had chosen the nursing profession and how their original perspectives on the profession may have changed since starting the program, supported by recollections of personal experiences.

We organized 8 focus group discussions with third-year nursing students of the Medical University of Bialystok, Poland. The selection of third-year students was motivated by the fact that they already had some experience of the profession and contact with nurses during the course and in professional traineeships. The rationale for choosing a focus group [27] as the most appropriate technique to perform this study was based on the following assumptions:

1. The homogeneous character of the group (participants of the same age and studying in the same year) ensures a sense of community, which facilitates open expression of ideas concerning the problems discussed, because the students have experienced the same challenges.
2. Interaction among the participants facilitates open communication concerning sensitive topics and promotes the expression of criticism of the profession, too.

According to Denzin and Lincoln [28], focus groups allow researchers to investigate ongoing group discourse in ways that individual interviews or observations do not permit. In our case, we hoped that the group dynamic would stimulate discussion among the participants, draw out opinions and ideas, and divulge new themes. The rationale for choosing the focus group technique in our study was to explore and describe the perception and choosing of the nursing profession from the perspective of students, expressed with their own vocabulary and in the context of their professional priorities.

### Interview procedure

All participants had voluntarily given informed consent before the start of the interview.

Each focus group involved 8–10 participants. A total of 76 students (Table 1) participated in the study; this number accounted for 95 % of all third-year nursing students of the Medical University of Bialystok in 2014. A trained moderator conducted all focus groups, using a semi-structured discussion guide (Additional file 1).

**Table 1:** Characteristics of participants (*n* = 76)

Focus Group	Number of participants	Age (in years)	Sex	
			Male	Female
1	10	21–23	0	10
2	10	20–30	0	10
3	10	21	0	10
4	10	21–24	0	10
5	10	21	0	10
6	8	21–22	0	8
7	10	21–24	1	9
8	8	21–25	0	8

Focus group discussions lasted 60 min on average; they were tape-recorded, and then transcribed by the first author (LM). All the focus groups had the same observer, the second author of the paper (AO), who observed the interactions between the participants.

Focus groups were conducted in Polish, the obtained data were also analyzed in Polish and then translated into English by a professional translator and then the translation was checked for correct interpretation of meaning by the last author (TP).

### Data analysis

The data were analyzed using thematic analysis. They were coded by theme and the key data representing each theme were extracted and subjected to inductive qualitative analysis [29] and open coding [30]. According to Patton, inductive analysis is “*discovering* patterns, themes, and categories in one’s data” ([29], p. 453). Open coding was performed independently by two authors (LM and AO). Transcripts from all focus groups were printed in two copies. The content of the transcripts was read several times and expressions referring to motivations for the choice of career were underlined in the text. Then the expressions were characterized and given names using key words. The coding results of the two researchers (LM and AO) were compared and in the cases of disagreement a solution was discussed with the third author (BS) until consent was reached. The analysis ended when no new information emerged during the coding and saturation of themes was achieved [30]. The categories had to correspond to the original text (students’ statements). To ensure credibility, we tried to identify representative quotations from the transcripts [31].

## Results

1. The reasons for choosing the nursing profession
  Data analysis revealed several reasons why students had chosen nursing as a career. These were: the desire to help others, family tradition, the desire to go abroad, failure to get into another course, pure chance, and low admission requirements (relative to medical studies). Some responses of the participants only referred to one subject/topic and were expressed as short sentences. For example, altruistic motivations associated with helping others were expressed like this:
  *“I want to help people who need assistance but cannot help themselves.”* (FG 7)
  There were also simple statements stressing that the desire to become a nurse was a continuation of the profession in the family.
  *“My mom used to work at the hospital. I’ve always had contacts with the hospital and I liked it.”* (FG 8)
  Some of the respondents were straightforward about choosing the nursing profession because it affords opportunities to work abroad.
  *“I chose the course because I want to go abroad. In other countries nursing is a good job, you are better treated, respected and well paid.”* (FG 2)
  The statements of some respondents, however, pointed to more complex motivations, a mixture of several reasons. On the one hand, students chose nursing for practical reasons, e.g. opportunities for finding a job. On the other hand, the decisions were affected by their family situation.
  *“In fact I applied to nursing and medical rescue. … Nursing made it possible to choose a specialization and gave me a broader range of job opportunities. But why nursing in the end? A few years before I went to university, my grandma got seriously ill and I spent a lot of time at the hospital and hospice; that situation kind of inspired me to do this. And besides, it was my mom’s dream. She wanted me to become a nurse … because in her opinion it’s a good job, and in the future I could help her if she needed it.”* (FG 4)
  Some utterances involved a mixture of other motivations for the choice of nursing, such as pure chance, failure to get into other courses, or family traditions.
  *“I was not admitted to psychology. I wanted to be a psychologist very much. … But I think it was not a random choice, because my parents are nurses. I suppose I chose nursing a bit subconsciously.”* (FG 3)
  Nursing was also chosen by people who evaluated their abilities as poor/low and were aware they would not be admitted to another course with higher requirements.
  *“In my case the decision was taken in secondary school. The girls from my class said that they would go to nursing, that you don’t need a very high number of points. So I thought I could try too. And during the studies I found that I like it very much, that it involves contact with people, that I will learn how to help others and I won’t be so passive.”* (FG 6)
  The statements by persons who chose nursing studies by accident were often negative.
  *“In my case it was just by accident. I was thinking about a medical course and finally I chose nursing. I was considering changing the course. But so far I’ve stayed here. I want to complete it. I took to it when I was in the second year. Before, I was ashamed to say I studied nursing. … Because it’s undervalued. You can even say it’s the worst occupation. I thought so in the beginning, before I got to know the job better.”* (FG 5)
  Some participants frankly admitted they would not choose the nursing profession again, because they did not feel fulfilled working as nurses. They explained it by saying that the decision to choose a future profession is taken too early and young people often do not know what they want to do in life.
  *“I wouldn’t choose nursing either. Everything I do at the hospital, I do casually. I just don’t feel well doing that. It’s as if it were a punishment. Whatever I do – a drip, an injection, whatever – I don’t feel like doing it! I know it’s not what I like. If I were to make a choice once again, I would choose something else. … When you are in the last form of secondary school, you are simply too young to make such serious decisions about what you want to do in life. Because you don’t know it yet. And later you regret.”* (FG 2)
  An idea present in some participants’ utterances was that, although they were aware of the often difficult and undervalued work of a nurse, they still chose the course of study hoping that the young generation of nurses could improve the image of the nurse in the community.
  *“From my experiences as a patient in hospital, I didn’t have any positive associations with nurses. I remember them to be rather unkind and insensitive. I would like both nurses’ attitudes toward patients and patients’ attitudes toward nurses to change. A nurse is obviously not perceived very well, she is not considered as important. But I think maybe if the staff are young, they will behave differently, maybe the image of a nurse will change a little, maybe she will be kinder and more sensitive for the patient.”* (FG 6)
  Motivation to study nursing is complex and, at least for some, it appears to be directly related to their perceptions of the profession, while for others there are more extrinsic factors.
2. Students’ perception of the nursing profession
  The participants’ images of the nursing profession were based on their personal experiences or observation of nurses’ work in various situations. Often these observations were superficial, only concerning selected fragments of nurses’ work.
  *“I imagine a nurse as a person working in a health care center, always smiling and drinking coffee. She may sometimes give someone an injection or take blood pressure, but that’s all.”* (FG 6)
  The participants’ utterances show that many young people had untrue images of the nursing job when they went to study this subject.
  *“In the beginning I didn’t know it’s such a hard job. I associated it with sitting in the staffroom, distributing drugs, taking blood samples, and that’s it, really. Now I know it’s a very hard and undervalued job.”* (FG 6)
  Students were not aware of what knowledge and skills are required of a nurse.
  *“For example, before I started the course, I thought the nurse’s job is one in which you don’t need such extensive knowledge and so many skills. I only learned at university how much work is needed to start doing this job. I thought I would study here and it would be so easy.”* (FG 7)
  The students realized that a nurse is not respected very much in the community, but their own self-perception of the position ‘improved’ because of the fact that nurses have to study, acquire considerable knowledge and learn many skills.
  *“In the beginning it was hard for me to accept this choice because I felt I had disappointed my parents. It seems in my family the nursing profession was not perceived as very ambitious. But going to the course and seeing how much studying was needed, I accepted this and as a matter of fact, I’m glad.”* (FG 3)
  Academic experiences of the participants let them see both negative and positive behaviors of nurses. They also had their own explanations for such attitudes of nurses.
  *“I think these are complexes arising from contacts with physicians. Because it is the doctor who is always regarded to save lives, treat patients and help. A nurse has always been on the sidelines, the least important, nobody used to pay attention to what she was doing. I think this is the reason why nurses have always felt undervalued. And this has continued from generation to generation, I would say a kind of internal anger. And later it is reflected in our contacts with them, but when you look ‘through the keyhole’ how they approach patients, you find they are great, they are cordial, kind, amusing and really caring. I don’t know, maybe sometimes they feel ashamed to be such good and cheerful persons.”* (FG 7)
  Some participants talked about the vocation one needs to do this job.
  *“I think there’s some vocation. It’s work, you have to do your tasks, but in my opinion it is one of the few jobs in which you need to give of yourself. If someone doesn’t do that and doesn’t care, that person definitely would not encourage others to do the job.”* (FG 6)
  But there were also some statements which denied the need for vocation in the nursing profession and pointed out the need for professionalism instead.
  *“I don’t think vocation really exists. In my opinion if a person chooses a job, they should try to do it professionally. For example, verbal or non-verbal communication with the patient should not be the result of the nurse’s vocation or the lack of it but of the fact that she knows it will help restore the patient’s health.”* (FG 6)
  Participants’ utterances show that the idealistic image of the nursing profession changes as a result of experiences gained during their practical education, when they observe first-hand the actual work that nursing entails. However, it seems that participants raise their self-esteem as future nurses by emphasizing the fact that they study at a university and devote large numbers of hours to that study.
3. Disrespect for the nursing profession from the participants’ point of view
  The participants identified several causes of the disrespect for the nursing profession.
  They thought nurses often contributed to the disrespect by their inappropriate attitudes and behaviors.
  *“I think one needs to deserve respect in order to be respected. … Some nurses lower the culture level of the whole group and then we say that nurses are nasty and rude.”* (FG 2)
  They observe that physicians sometimes lower the prestige of the occupation when the doctor comments on the tasks of future nurses in the company of patients.
  *“Even the doctors treat us poorly. When we had our professional traineeships, we were in the room and were talking to the patient, the doctor told us to wash the patient because we were there for that reason, weren’t we? … Patients can hear this and then they perceive us this way, that a nurse is always lower than a doctor.”* (FG 1)
  For them, the problem was that doctors did not know enough about the range and variety of tasks a nurse does.
  *“Doctors often do not respect nurses either. They seem not to see really how much a nurse must do in the ward before the doctor comes to work. He does not see what happens between 7 and 9 a.m., when the nurse must do everything by herself.”* (FG 5)
  In the participants’ opinion, doctors’ negative attitude toward nurses has been consolidated for years and may result from family traditions.
  “*It seems to me that you acquire this attitude in your family, because doctors usually come from families of doctors*.” (FG 5)
  The participants also saw the source of the disrespect in the role-conflict between physicians and nurses and in the lack of cooperation. Yet, they were of the opinion that both doctors and nurses were to blame for this.
  “*There is this eternal conflict between doctors and nurses. … Doctors will always be on a higher level, and nurses will always be on the level they are on now, even if they have higher education. We do what they tell us to do. The orders come top down. No respect, no cooperation. But it’s not always like this. There are some wards where you can see some cooperation with the physicians. This depends both on them and on us.*” (FG 2)
  The participants presented a few proposals of how to increase the respect for the job of a nurse. Some of them thought the respect for the profession depends on the nurses themselves.
  *“Nurses should respect each other, and often there are arguments within the staff.”* (FG 5)
  They suggested nurses should demonstrate greater activity in autonomous professional tasks.
  *“A nurse should take the initiative to a greater extent, not only do what she is told by the physician.”* (FG 4)
  The need to educate the community regarding nurses’ knowledge, skills and university education was also emphasized.
  *“Many people know that future doctors study at medical universities, but they do not know nurses study there as well. My grandma would introduce me to someone and say ‘My granddaughter is a student of nursing’. The surprise was great! Can you study this at university? Because there’s a stereotype that at a medical university you can study to become a doctor but not to become a nurse.”* (FG 4)
  Another proposal was for doctors and nurses to meet together so as to learn about the tasks done by each other, especially the varied tasks of a nurse.
  *“Doctors and nurses could meet to allow each of them to see the range of responsibilities of the other group, for doctors to see that a nurse does much more than what she is told to do by the doctor. She has many more responsibilities. She has to support the patients and talk to them.”* (FG 4)
  They also underscored that respect for the nursing profession is related to adequate pay.
  *“Nurses are poorly paid. They go on strike from time to time because they want to earn more. People think that if they earn little, it means they know little and do little, too.”* (FG 3)
  According to participants, the lack of respect for the nursing profession is the result of many factors, such as mutual relationships between nurses, the lack of understanding of the roles of nurses among physicians, public opinion, and financial matters.

## Discussion

The applied methodology (focus groups) made it possible to investigate the complexity of students’ choice and perception of the nursing profession. The complex character of the motivating forces which propelled the young Polish students (nearly all women) in our study to train as nurses has also been described by other investigators in Poland. For example, the findings of Dziubak & Motyka [22] highlight the mixed character of motivations for the choice of nursing education by students at a different institution from ours, with a clear dominance of pro-community motivations focused on the good of others. Our research also shows negative motivations, namely that young people sometimes choose the nursing profession without full awareness of what it involves. Students have a different view of nurses’ work before starting the university program, and during the coursework it is possible to encourage the less-committed students by promoting greater reflection on their individual and collective roles as nurses. Other studies show that the system of education has an impact on the nurses’ qualifications and their image defined as the level of knowledge, skills and attitudes [32]. A Polish study by Slusarska et al. demonstrates that the type of education (1^st^ and 2^nd^ level of occupational differentiation) has a statistically significant effect on self-definition of nursing in terms of the nature of the occupational activities performed [33].

The participants’ statements show that, even during their studies, student nurses experience disrespect for the nursing profession and are aware of the difficult conditions in which they will work in the future. For instance, a continuing problem for Polish nursing is the shortage of support staff. The new profession of “health care assistant” was introduced mainly for the purpose of long-term home care, not hospital care, and is not a solution to this problem [3]. The study participants also offered some suggestions about how to improve the image of a nurse, although they realize it is hard to change society’s perception of the job which for so many years has been perceived as merely ancillary to doctors’ work. The process of changing commonly held views is a long-term one. It seems, however, that the contemporary higher education now required of the nursing profession in Poland is enabling the students to think critically about their discipline and the importance of their work.

Interactions among the participants during the group discussion produced data which present both positive and negative aspects of the profession. During the discussion, the participants gave mutual encouragement, which made them feel free to express criticism, too. This allowed us to obtain data which could never be generated using other research methods. For example, although vocation is a desired quality reported by nurses-to-be [19], the findings presented here suggest that the new generation of future Polish nurses prefers to stress professionalism in this occupation.

The participants see chances for increasing respect for the nursing profession both in their own attitudes and behaviors as nurses-to-be, in organizational matters (e.g. by raising remuneration), and in better cooperation with physicians. A cross-sectional study of the nursing profession conducted among Polish medical students confirmed that they mostly perceive nurses in terms of the tasks associated with the therapeutic process rather than the development of independence. Polish medical students mainly perceive nurses through skills and abilities such as professional reliability, good technical skills and being friendly and courteous [34].

The problems of doctor-nurse cooperation found in this original study are also confirmed in the international literature [35]. Sharing of learning experiences between nursing and medical students might improve understanding of the respective roles, which is supported by the findings of a systematic review [36]. Results obtained by other authors show that interprofessional education leads to improved self-reported effectiveness as an interprofessional team member, and self-perceived knowledge and ability to collaborate with others to improve patient outcomes [37]. The lack of mutual respect among nurses, pointed out by the participants in our study, has also been reported by scholars studying Iranian nurses [38].

Another aspect that needs to be stressed in the Polish socio-occupational situation of young people deciding on their future career, is the perception of nursing in terms of its ability to ensure job security and permanent income (albeit the latter currently low). Our research shows how important this perspective is for the participants, mainly by their statements that they plan to work abroad in the EU. To counteract this, efforts are needed to promote the nursing profession and to create the conditions conducive to nurses staying in Poland.

It must be pointed out that our participants, apart from one man, are female students, because in Poland the nursing profession is female-dominated. This fact may have an influence on the participants’ perception of the job.

## Conclusions

Within the scope of medical professions, the decision about nursing is mainly determined by practical aspects, e.g., the opportunity to find a job. Although young people are aware of the low prestige of the nursing profession in Poland, they believe it is possible to improve its image and enhance its prestige. Students can see the chance for improvement of the nursing profession by means of accreditation requiring professional preparation at the university level. During the university course, nursing students point out the need to enhance the esteem in this profession. Both medical universities/schools and nursing managers should create an authentic and up-to-date image of the nursing occupation, presenting its advantages and disadvantages, in order to attract prospective students to the profession.

## Additional file

Additional file 1: The focus group guide. (DOC 42 kb)
