# Supplementary material for: Choice and perception of the nursing profession from the perspective of Polish nursing students: a focus group study
Source: BMC Med Educ. 2016 Sep 20;16:243. doi: 10.1186/s12909-016-0765-3 (PMC5029103; doi:10.1186/s12909-016-0765-3)
Supplement: Additional file 1: — The focus group guide. (DOC 42 kb) [file 12909_2016_765_MOESM1_ESM.doc]

Additional file 1: The focus group guide.

1. Why did you choose to study nursing?
2. What are your experiences with the job of a nurse during your university education?
3. Has the university course in nursing met your expectations?

- Why do you think the nursing profession is not respected?

1. Would you choose the nursing profession again?

- Why wouldn't you choose the nursing profession again?

1. Would you like your children to choose the job as a nurse?
